# Real-world patient characteristics, treatment patterns, and clinical outcomes associated with tucatinib therapy in HER2-positive metastatic breast cancer

**DOI:** 10.3389/fonc.2023.1264861

**Published:** 2023-10-02

**Authors:** Peter A. Kaufman, Edward Neuberger, Naomi R. M. Schwartz, Shu Wang, Yutong Liu, Ling-I Hsu, Karen Bartley, Matthew T. Blahna, Brian T. Pittner, Gabriel Wong, Carey Anders

**Affiliations:** ^1^ Division of Hematology and Oncology, University of Vermont Medical Center, Burlington, VT, United States; ^2^ Seagen Inc., Bothell, WA, United States; ^3^ Genesis Research, Hoboken, NJ, United States; ^4^ Division of Medical Oncology, Duke Cancer Institute, Durham, NC, United States

**Keywords:** metastatic breast cancer, HER2+, brain metastases, tucatinib, real-world

## Abstract

**Background:**

Tucatinib is an oral human epidermal growth factor receptor 2 (HER2)-directed therapy approved in combination with trastuzumab and capecitabine for use in patients with previously treated HER2+ metastatic breast cancer (MBC) with/without brain metastases (BM). To inform clinical decision-making, it is important to understand tucatinib use in real-world clinical practice. We describe patient characteristics, treatment patterns, and clinical outcomes for tucatinib treatment in the real-world setting.

**Methods:**

This retrospective cohort study included patients diagnosed with HER2+ MBC (January 2017-December 2022) who received tucatinib treatment in a nationwide, de-identified electronic health record–derived metastatic breast cancer database. Patient demographics and clinical characteristics were described at baseline (prior to tucatinib initiation). Key outcomes included real-world time to treatment discontinuation (rwTTD), time to next treatment (rwTTNT), and overall survival (rwOS).

**Results:**

Of 3,449 patients with HER2+ MBC, 216 received tucatinib treatment (n=153 with BM; n=63 without BM) and met inclusion criteria. Median (range) age of patients was 56 (28-84) years, 57.9% were White, and 68.5% had Eastern Cooperative Oncology Group performance status ≤1. Median (IQR) follow-up from start of tucatinib treatment was 12 (6-18) months. Among all patients who received tucatinib treatment, median (95% CI) rwTTD was 6.5 (5.4-8.8) months with 39.8% and 21.4% remaining on treatment at 12 and 24 months, respectively. Median (95% CI) rwTTNT was 8.7 (6.8-10.7) months. Patients who received the approved tucatinib triplet combination after ≥1 HER2-directed regimen in the metastatic setting had a similar median (95% CI) rwTTD (any line: 8.1 [5.7-9.5] months; second-line (2L) and third-line (3L): 9.4 [6.3-14.1] months) and rwTTNT (any line: 8.8 [7.1-11.8] months; 2L and 3L: 9.8 [6.8-14.1] months) to the overall population. Overall, median (95% CI) rwOS was 26.6 (20.2-not reached [NR]) months, with similar findings for patients who received the tucatinib triplet (26.1 [18.8-NR] months) and was NR in the subgroup limited to the 2L/3L population.

**Conclusion:**

Tucatinib treatment in the real-world setting was associated with a similar median rwTTD, rwTTNT, and rwOS as in the pivotal HER2CLIMB trial, with particular effectiveness in patients in the 2L/3L setting. These results highlight the importance of earlier use of tucatinib in HER2+ MBC.

## Introduction

Breast cancer is the most commonly diagnosed cancer among women and is a leading cause of cancer death, with an estimated 2.3 million new cases worldwide in 2020 (1). Approximately 15% and 26% of patients diagnosed with early stage and metastatic breast cancer (MBC), respectively, have human epidermal growth factor receptor 2 (HER2)-positive (HER2+) tumors (2). HER2+ MBC has an aggressive clinical phenotype and has historically been characterized by a high rate of recurrence and poor survival (3). In addition, up to 50% of patients with HER2+ MBC develop brain metastases over the course of their disease, a development associated with treatment complications and worse duration of survival (4, 5).

Tucatinib is a highly selective, oral tyrosine kinase inhibitor (TKI) of the HER2 receptor that exerts minimal inhibition of epidermal growth factor receptor (EGFR) (6, 7). Recently, tucatinib was approved in combination with trastuzumab and capecitabine in multiple countries for adult patients with HER2+ MBC, including patients with brain metastases (8). These approvals were based on results of HER2CLIMB, a randomized, double-blinded clinical trial which evaluated the efficacy and safety of tucatinib vs placebo, each in combination with trastuzumab and capecitabine in patients with previously treated HER2+ MBC (9). In the study, the combination of tucatinib, trastuzumab, and capecitabine demonstrated statistically significant and clinically meaningful improvements in progression-free survival (PFS) (7.8 vs 5.6 months; hazard ratio (HR): 0.54, 95% CI: 0.42-0.71) and overall survival (OS) (21.9 vs 17.4 months; HR: 0.66, 95% CI: 0.50-0.88) with a manageable safety profile (9). Uniquely, HER2CLIMB included patients with progressing brain metastases, a population normally excluded from clinical trials due to the poor treatment outcomes in this setting.

While HER2-directed therapies remain the mainstay of systemic treatment for HER2+ metastatic breast cancer, the treatment landscape is rapidly evolving, with multiple new HER2-targeted treatments emerging in recent years. In addition to tucatinib, several new HER2-targeted therapies, including another TKI, a novel monoclonal antibody, and an antibody drug conjugate (ADC), have been approved since 2020 in the US. Of these, tucatinib, trastuzumab, and capecitabine, as well as the ADC trastuzumab deruxtecan (T-DXd), are currently recommended in the 2L and 3L setting in US and European guidelines, replacing previous standards such as the ADC trastuzumab emtansine (T-DM1) in second-line (2L) and multiple options including trastuzumab + capecitabine and lapatinib + capecitabine in third-line (3L) (10, 11). Due to the pace of development in this disease space, treatment patterns and outcomes for patients receiving these combinations in the real world setting are unknown.

To better inform clinical decision-making, it is important to understand tucatinib use and performance in real-world clinical practice. This retrospective study describes characteristics, treatment patterns, and clinical outcomes for patients with HER2+ MBC with and without brain metastases treated with tucatinib regimens in the real-world setting.

## Methods

### Study design and data source

This retrospective cohort study included patients diagnosed with HER2+ MBC from January 2017 to December 2022 who received tucatinib treatment in a nationwide, deidentified electronic health record–derived Flatiron Health metastatic breast cancer database. At the time of the study, the data were derived from >280 US cancer clinics (~800 sites of care). The Flatiron Health database includes structured data (eg, demographics, laboratory values, prescribed drugs) and unstructured data (eg, biomarker levels) collected via technology-enabled chart abstraction from physicians’ notes and other unstructured documents (12, 13). Institutional review board approval was not required, as the study was noninterventional, and only deidentified patient records were used.

### Patient population

The study included patients aged ≥18 years diagnosed with MBC between January 1, 2017 and December 31, 2022 who had evidence of HER2-receptor positivity (defined as either fluorescence *in-situ* hybridization positive, next-generation sequencing positive, positive not otherwise specified, or immunohistochemistry 3+) prior to or within 90 days of MBC diagnosis. Exclusion criteria included a lack of systemic treatment in the metastatic setting, no activity in the Flatiron Health database within 90 days of MBC diagnosis, and evidence of previous non–breast cancer within the 6 months prior to MBC diagnosis. Included patients who received HER2-directed therapies were evaluated in the following analysis cohorts (not mutually exclusive): (1) patients who received any tucatinib treatment in any line of therapy (LOT), (2) patients who received the US Food and Drug Administration (FDA)–approved tucatinib triplet combination (tucatinib in combination with trastuzumab and capecitabine) after receiving ≥1 HER2-directed therapies in the metastatic setting, (3) patients who received the FDA–approved tucatinib triplet combination in 2L or 3L following ≥1 prior HER2-directed therapies in the metastatic setting, and (4) patients who received tucatinib treatment immediately following T-DXd in any LOT. Outcomes for group 1 and 2 were also stratified by LOT. The presence of brain metastases prior to tucatinib initiation was determined via chart-abstracted data.

### LOT definitions

The first oncologist-defined, rule-based LOT began on the first administration or abstracted therapy occurring on or after metastatic diagnosis. All treatments that began within 28 days of the first treatment date were considered part of the same LOT. Where physician-indicated end date was not provided, treatment discontinuation was defined as a treatment gap of >60 days from the last medication date for a regimen. A treatment switch was defined as a claim for a new HER2-directed therapy, TKI, or chemotherapy ≥29 days after the line start date. The addition of a hormone therapy was deemed unlikely to be due to disease progression and so was not considered when defining LOT.

### Outcomes

Objectives of this study were to describe baseline demographic and clinical characteristics, treatment patterns (including sequencing), and clinical outcomes for patients with HER2+ MBC receiving tucatinib regimens. Clinical outcomes included were real-world time to treatment discontinuation (rwTTD; time from treatment initiation to discontinuation), persistence (proportion of patients remaining on therapy at 6, 12, 18, and 24 months), real-world time to next treatment (rwTTNT) as a proxy for PFS (14, 15) (time from treatment initiation to the start of the subsequent LOT), and real-world overall survival (rwOS; time from treatment initiation to death).

### Statistical methods

Demographic and clinical characteristics were assessed during the baseline period (prior to initiation of tucatinib regimens). Patients were followed until either the end of the study period, loss to follow-up, or death, whichever occurred first. Continuous variables were described using mean, standard deviation (SD), median, and interquartile range (IQR). Categorical variables were described using frequency (number of cases) and percentage of total patients observed in each category. Time-to-event analyses were conducted for rwOS, rwTTNT, and rwTTD using the Kaplan–Meier method. Patients without recorded events were censored at their last recorded activity date. Persistence was defined as the proportion of surviving patients with sufficient follow-up remaining on tucatinib therapy at 6, 12, 18, and 24 months. All analyses were conducted using SAS 9.4 and R 4.2.0.

## Results

### Patient characteristics

Of 32,819 patients with an MBC diagnosis between January 1, 2017 and December 31, 2022, 3,449 had evidence of HER2+ disease. Of these patients, 216 received tucatinib treatment and met all inclusion and exclusion criteria (**Supplementary Table 1**). Median (range) age of included patients was 56 (28–84) years, 57.9% were White, and 68.5% had Eastern Cooperative Oncology Group performance status (ECOG PS) ≤1 (**Table 1**). Median (IQR) follow-up from the start of tucatinib treatment was 12 (6–18) months.

**Table 1:** Baseline characteristics^a^ for patients with HER2+ MBC receiving tucatinib therapy in the EHR-derived database.

Characteristic	All patients (N=216)
Age (years), median (range)	56 (28–84)
Race, n (%)	
White	125 (57.9)
Black	33 (15.3)
Asian	6 (2.8)
Other	38 (17.6)
Unknown	14 (6.5)
*De novo* MBC diagnosis, n (%)	89 (41.2)
ECOG PS, n (%)	
0	65 (30.1)
1	83 (38.4)
2+	21 (9.7)
Missing	47 (21.8)
Site of metastasis,^b^ n (%)	
Brain	153 (70.8)
Bone	137 (63.4)
Liver	98 (45.4)
Lung	93 (43.1)
Prior lines of therapy, median (range)	2 (0-10)
0	23 (10.6)
1	69 (31.9)
2	44 (20.4)
3+	80 (37.0)

a Baseline refers to the period prior to tucatinib treatment initiation.

b Not mutually exclusive.

ECOG PS, Eastern Cooperative Oncology Group performance status; EHR, electronic health record; HER2, human epidermal growth factor receptor 2; MBC, metastatic breast cancer.

**Figure 1:**
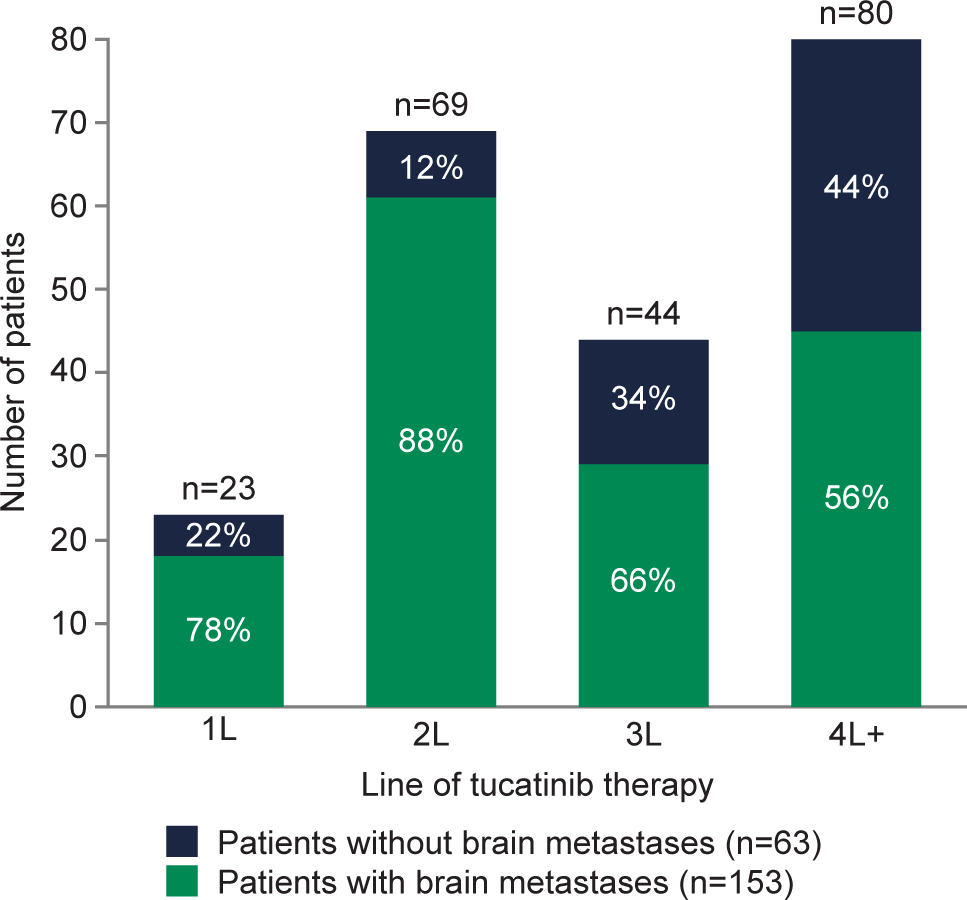
Presence of brain metastases prior to tucatinib initiation among patients with HER2+ MBC in the electronic health record (EHR)-derived database by LOT. 1L, first-line; 2L, second-line; 3L, third-line; 4L+, fourth-line and beyond; EHR, electronic health record; HER2, human epidermal growth factor receptor 2; LOT, line of therapy; MBC, metastatic breast cancer.

Of the 216 patients who received tucatinib treatment, prior to treatment initiation, 70.8% (n=153) had brain metastases (**Figure 1**) and 64.8% (n=140) had visceral metastasis (**Supplementary Table 2**). Median (IQR) lines of prior therapy was 2 (1–3) among all patients and 1 (1–3) and 3 (2–4) among patients with and without brain metastases, respectively (**Supplementary Table 2**). Compared with patients without brain metastases prior to tucatinib treatment, those with brain metastases were younger (53 vs 62 years) and had a higher proportion of involvement of 3+ metastatic sites (69.9% vs 42.9%). Patients with brain metastases had a shorter duration of time between MBC diagnosis and start of tucatinib therapy (19 vs 26 months), and a lower proportion received prior T-DXd (7.2% vs 47.6%) and T-DM1 (41.2% vs 73.0%) (**Supplementary Table 2**).

### Treatment patterns

Most patients (n =159; 73.6%) received tucatinib in combination with trastuzumab and capecitabine in any line, and 144 (66.7%) patients received the tucatinib triplet after ≥1 prior anti-HER2 regimen in the metastatic setting. Of 159 patients initiating tucatinib triplet therapy in any line, 16 (10.1%) discontinued capecitabine ≥1 month prior to discontinuing tucatinib therapy. Of the 57 patients who did not receive tucatinib, trastuzumab, plus capecitabine, 26 (45.6%) patients received tucatinib with trastuzumab, 15 (26.3%) received tucatinib with capecitabine, 8 (14.0%) received tucatinib monotherapy, and 8 (14.0%) received other tucatinib combinations. The most common regimen prior to 2L tucatinib treatment was trastuzumab plus pertuzumab (51/69, 73.9%), and the most common regimens immediately following 2L tucatinib treatment were T-DXd (18/33, 54.6%) and trastuzumab (10/33, 30.3%). The most common regimens immediately prior to and following 3L tucatinib treatment were T-DM1 (27/44, 61.4%) and T-DXd (19/28, 67.9%), respectively.

### rwTTD, persistence, and rwTTNT

**Figure 2:**
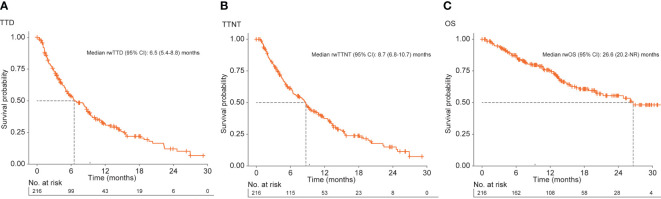
Kaplan–Meier curves for **(A)** rwTTD, **(B)** rwTTNT, and **(C)** rwOS among patients with HER2+ MBC receiving tucatinib regimens in the EHR-derived database. EHR, electronic health record; HER2, human epidermal growth factor receptor 2; MBC, metastatic breast cancer; NR, not reached; OS, overall survival; rw, real world; TTD, time to discontinuation; TTNT, time to next treatment.

Median (95% CI) TTD was 6.5 (5.4-8.8) months for all patients and was longer for patients who received the approved tucatinib triplet combination after receiving ≥1 prior anti-HER2-based regimen in the metastatic setting (8.1 [5.7-9.5] months) or in 2L and 3L (9.4 [6.3-14.1] months) (**Table 2**; **Figure 2A**). Of the patients receiving tucatinib treatment with sufficient follow-up, 43 of 108 (39.8%) were still receiving tucatinib at 12 months, and 6 of 28 (21.4%) were still receiving tucatinib at 24 months (**Table 2**). For all patients receiving tucatinib treatment, rwTTD was similar across earlier LOT (first-line [1L] to 3L: 8.2-9.4 months), with a decrease observed in fourth-line and beyond (4L+) (4.8 months). A similar pattern was observed for patients receiving the tucatinib triplet combination after ≥1 prior anti-HER2-based regimens in the metastatic setting (2L, 3L, and 4L+: 8.6, 9.4, and 5.4 months, respectively) (**Supplementary Table 3** **)**.

**Table 2:** Median rwTTD, rwTTNT, and rwOS among patients with HER2+ MBC receiving tucatinib regimens in the EHR-derived database.

	**All patients** **(N=216)**	Approved tucatinib triplet combination in any line^a^ **(n=144)**	**Approved tucatinib triplet combination in** 2L and 3L^b^ **(n=83)**
rwTTD (months), median, (95% CI)	6.5 (5.4-8.8)	8.1 (5.7-9.5)	9.4 (6.3-14.1)
Persistence,^c^ % (n/N)			
6 months	61.1 (99/162)	61.3 (68/111)	67.7 (44/65)
12 months	39.8 (43/108)	40.0 (28/70)	42.2 (19/45)
18 months	29.3 (17/58)	27.0 (10/37)	26.1 (6/23)
24 months	21.4 (6/28)	10.5 (2/19)	14.3 (2/14)
rwTTNT (months), median, (95% CI)	8.7 (6.8-10.7)	8.8 (7.1-11.8)	9.8 (6.8-14.1)
rwOS (months), median, (95% CI)	26.6 (20.2-NR)	26.1 (18.8-NR)	NR

a Patients who received the FDA-approved tucatinib triplet combination (tucatinib in combination with trastuzumab and capecitabine) after receiving ≥1 HER2-directed therapies in the metastatic setting.

b Patients who received the FDA-approved tucatinib triplet combination in 2L or 3L following ≥1 prior HER2-directed therapies in the metastatic setting.

c Proportion of patients with follow-up and still on therapy at 6, 12, 18, and 24 months.

2L, second-line; 3L, third-line; EHR, electronic health record; HER2, human epidermal growth factor receptor 2; MBC, metastatic breast cancer; OS, overall survival; NR, not reached; rw, real world; TTD, time to discontinuation; TTNT, time to next treatment.

Median (95% CI) rwTTNT was 8.7 (6.8-10.7) months for all patients (**Table 2**; **Figure 2B**) and was similar for patients who received the approved tucatinib triplet regimen in any line (8.8 [7.1-11.8] months) or in the 2L and 3L setting (9.8 [6.8-14.1] months). Among all patients who received tucatinib treatment, the proportion (95% CI) of patients who had not initiated a subsequent HER2-directed treatment following tucatinib was 37.2% (30.7-45.0) at 12 months and 14.9% (9.4-23.5) at 24 months. Median rwTTNT for all patients decreased with increasing LOT from 12.8 months in 1L to 6.5 months in 4L+. This trend was also seen among patients who received the tucatinib triplet combination (2L: 11.0 months; 4L+: 8.1 months) (**Supplementary Table 3** **)**.

### rwOS

For all patients who received any tucatinib therapy, median (95% CI) rwOS was 26.6 (20.2-not reached [NR]) months. This was lower for patients receiving tucatinib in the 4L+ setting (median [95% CI] = 16.6 [12.2-NR]) (**Supplementary Table 3**). Median (95% CI) rwOS among patients who received the tucatinib triplet combination in any line was 26.1 (18.8-NR) and NR in the subgroup limited to the 2L and 3L patient population (**Table 2**, **Figure 2C**). Overall, 12- and 24-month survival probabilities (95% CI) were 75.3% (69.3-81.9) and 55.5% (47.3-65.0), respectively. Among patients initiating the tucatinib triplet combination, 12- and 24-month survival probabilities (95% CI) were 75.9% (68.4-84.1) and 54.7% (44.7-66.9), respectively, for those receiving the regimen in any line and 85.0% (76.7-94.2) and 63.8% (50.5-80.7), respectively, for patients receiving the combination in 2L and 3L.

### rwTTD, rwTTNT, and rwOS for the tucatinib post–T-DXd subgroup

Thirty-five patients received tucatinib treatment immediately following T-DXd (**Supplementary Table 4** **)**. Similar to the overall population, median (range) age of patients in this subgroup was 59 (36–84) years, and the majority (77.1%) of patients had ECOG PS of ≤1. Median (IQR) follow-up from the start of tucatinib treatment was 7.1 (4.1-13.5) months among the entire subgroup and 6.6 (5.0-14.2) months and 7.8 (3.4-13.4) months among patients with brain metastases (n=7) and without brain metastases (n=28) pre-tucatinib, respectively. These patients were heavily pretreated, and median LOT for tucatinib for this subgroup was 4, with 30 (85.7%) receiving ≥3 prior lines of therapy.

For T-DXd therapy received prior to tucatinib, median (95% CI) rwTTD and rwTTNT was 4.6 (3.7, 10.3) months and 5.4 (4.4-10.6) months, respectively. For patients who received tucatinib following T-DXd, median (95% CI) rwTTD was 6.4 (3.5-9.4) months, rwTTNT 8.1 (4.5-11.5) months, rwOS 13.9 (12.2-NR) months, and 12-month survival probability 69.2% (53.7-89.0).

## Discussion

With the emergence of multiple effective HER2-targeted treatment options, including tucatinib, the HER2+ MBC treatment landscape is changing rapidly. Decisions on the optimal sequence in which to use these emerging therapies in clinical practice should take into account how they perform in the real-world setting. Tucatinib was approved in combination with trastuzumab and capecitabine for previously treated HER2+ MBC based on the strength of the HER2CLIMB trial, a global, randomized, pivotal trial designed to reflect a clinically relevant patient population (eg, by including patients with brain metastases) (9). The current retrospective study described characteristics, treatment patterns, and clinical outcomes for patients diagnosed with HER2+ MBC who received tucatinib treatment in the real-world setting. The patient population receiving tucatinib in this study was similar to the HER2CLIMB trial population, though patients tended to present with poorer performance status (10% had an ECOG PS of 2+ while all patients enrolled in HER2CLIMB had an ECOG PS of ≤1) and had a greater prevalence of brain (71% vs 48%), bone (63% vs 54%), and liver (45% vs 33%) metastases. Patients in this study also had greater racial diversity (over one-third of patients were non-White compared with 29.7% in HER2CLIMB) and fewer prior lines of therapy (2 vs 3).

Despite these differences, this real-world analysis confirms the efficacy results of HER2CLIMB. Tucatinib treatment was associated with a similar or higher median (95% CI) rwTTD (6.5 [5.4-8.8] months), rwTTNT as a proxy for PFS (14, 15) (8.7 [6.8-10.7] months), and rwOS (26.6 [20.2-NR] months) compared with HER2CLIMB (duration of tucatinib exposure: 7.3 [range:<0.1 to 35.1] months; PFS: 7.8 [7.5-9.6] months; OS: 21.9 [18.3-31.0] months) (9). The rwOS findings from our study, where almost two-thirds of patients had visceral metastases, were also comparable with that found for the HER2CLIMB exploratory analysis in patients with visceral metastases (n=455; median [95% CI] OS: 21.6 [18.1-25.6] months). These data reinforce tucatinib’s durable effectiveness in the real-world setting in patients with HER2+ MBC with or without brain metastases. Importantly, this effectiveness was modestly enhanced by use of the tucatinib triplet combination in the 2L or 3L setting, consistent with the FDA label and its listing in various guidelines (10, 11).

These findings are noteworthy given that data from real-world experience often do not reflect clinical trial outcomes (16). A growing body of literature has highlighted an “efficacy-effectiveness gap” defined as the difference between outcomes within randomized clinical trials and those observed in real-world studies (16–20). This phenomenon has been noted for new treatments across various cancer types including HER2+ MBC (21–23). In a population-based retrospective cohort study in patients with HER2+ MBC, a gap between efficacy and effectiveness was identified for the HER2-directed therapies pertuzumab and T-DM1. Survival outcomes associated with pertuzumab and T-DM1 (median OS: 43 and 15 months, respectively) in the real-world setting were considered inferior to results from the pivotal clinical trials (median OS: 57 and 30 months for pertuzumab and T-DM1, respectively) (24). In our study, the comparability of the real-world effectiveness outcomes (rwTTD, rwTTNT as a proxy for PFS, and rwOS) and results from the pivotal HER2CLIMB clinical trial (9) highlight the use of tucatinib as a viable treatment option in the 2L and 3L setting for patients with HER2+ MBC.

Based on results from the DESTINY-Breast03 study (25), T-DXd has emerged as the standard treatment option in the 2L setting for patients without brain metastases (26). Because it was so recently approved, there are no data from randomized controlled trials on the efficacy of other HER2-targeting agents following treatment with T-DXd. We examined the effectiveness of tucatinib in the subset of patients (n=35) who received prior T-DXd. While small, the patient population was sufficient to demonstrate meaningful anti-tumor activity following T-DXd. Effectiveness (rwTTD, rwTTNT, and rwOS) in this group was numerically lower than for the overall patient population; however, the results were comparable with those for all patients who received tucatinib in the 4L+ setting. This finding is consistent with the fact that, on average, patients treated with prior T-DXd received tucatinib in a later line and at a longer time since metastatic diagnosis than for the overall patient population. These findings are especially compelling because these patients had a shorter duration of T-DXd therapy in this study than described in either DESTINY-Breast02 or 03.

Further studies will help determine the optimal positioning of tucatinib in the treatment pathway for HER2+ MBC, including how best to combine tucatinib with other HER2-directed therapies. The prospective, randomized HER2CLIMB-02 trial will evaluate the efficacy and safety of tucatinib plus T-DM1 vs T-DM1 and placebo in patients with HER2+ MBC progressing after pertuzumab and trastuzumab (27). The phase 3, randomized, double-blind HER2CLIMB-05 trial will assess efficacy and safety outcomes following the addition of tucatinib to 1L standard of care as maintenance therapy for HER2+ MBC (28).

### Limitations

While the study period was selected to reflect current clinical practice in a rapidly evolving treatment landscape, it excluded patients diagnosed with metastatic disease prior to 2017 who may have received tucatinib in later lines of therapy (ie, 4L+) and who likely had poorer clinical outcomes. Additionally, due to the recency of T-DXd approval, patients with a shorter duration of T-DXd prior to tucatinib therapy were more likely to be included in our study than those who achieved better outcomes on T-DXd therapy, limiting the generalizability of the post**–**T-DXd subgroup analysis. This evolving treatment landscape may also impact comparisons with prior clinical trials as new therapies have become available to patients in this study that were not available during recruitment to the HER2CLIMB clinical trial. Findings of the full analysis also may not be generalizable to patient populations not represented in the Flatiron Health database. Data on progression and reasons for discontinuation were not available in our dataset, so initiation of subsequent therapies was used as a proxy for progression (14) as is common with real-world data. It is possible that treatment changes were due to reasons other than progression (eg, toxicity or patient preference) or that patients had isolated brain metastasis progression that was treated with local therapy without a change in systemic therapy. If the former, our results could underestimate the true effectiveness of tucatinib, while the latter scenario would result in an overestimation. However, given the opposing directions of these two potential causes of misclassification, it is reasonable to expect that any bias would be minimal. All analyses in this study were unadjusted and the comparisons were purely descriptive. There may be differences in clinical or demographic characteristics across treatment groups that explain some of the variations in outcomes. Sample sizes were too small to stratify both by brain metastases status and LOT. Finally, as is true for all real-world data studies, there is risk of bias due to missing or incomplete data.

## Conclusions

Clinical outcomes among patients with HER2+ MBC who received tucatinib in real-world practice were similar to those observed in the HER2CLIMB trial, with effectiveness also observed among a small cohort of patients previously treated with T-DXd. Tucatinib regimens demonstrated effectiveness across all lines of therapy in a real-world setting in patients with HER2+ MBC with and without brain metastases.

## Data availability statement

The original contributions presented in the study are included in the article/**Supplementary Material**. Further inquiries can be directed to the corresponding author.

## Ethics statement

Ethical approval was not required for the study involving humans in accordance with the local legislation and institutional requirements. Written informed consent to participate in this study was not required from the participants or the participants’ legal guardians/next of kin in accordance with the national legislation and the institutional requirements.

## Author contributions

PAK: Conceptualization, Methodology, Writing- original draft, Writing- review & editing.. EN: Conceptualization, Methodology, Writing- original draft, Writing- review & editing. NRMS: Conceptualization, Methodology, Writing- original draft, Writing- review & editing. SW: Conceptualization, Methodology, Data curation, Formal analysis, Writing- original draft, Writing- review & editing. YL: Conceptualization, Methodology, Data curation, Formal analysis, Writing- original draft, Writing- review & editing. L-IH: Conceptualization, Methodology, Writing- original draft, Writing- review & editing. KB: Conceptualization, Methodology, Writing original draft, Writing- review & editing. MTB: Conceptualization, Methodology, Writing- original draft, Writing review & editing. BTP: Conceptualization, Methodology, Writing- original draft, Writing- review & editing. GW: Conceptualization, Methodology, Writing- review & editing. CA: Conceptualization, Methodology, Writing- original draft, Writing- review & editing.

## References

[1] Sung H Ferlay J Siegel RL Laversanne M Soerjomataram I Jemal A. Global Cancer Statistics 2020: GLOBOCAN Estimates of Incidence and Mortality Worldwide for 36 Cancers in 185 Countries. CA Cancer J Clin (2021) 71(3):209–49. doi: 10.3322/caac.21660 33538338

[2] Gong Y Liu YR Ji P Hu X Shao ZM. Impact of molecular subtypes on metastatic breast cancer patients: a SEER population-based study. Sci Rep (2017) 7:45411. doi: 10.1038/srep45411 28345619 PMC5366953

[3] Loibl S Gianni L. HER2-positive breast cancer. Lancet (2017) 389(10087):2415–29. doi: 10.1016/S0140-6736(16)32417-5 27939064

[4] Brufsky AM Mayer M Rugo HS Kaufman PA Tan-Chiu E Tripathy D. Central nervous system metastases in patients with HER2-positive metastatic breast cancer: incidence, treatment, and survival in patients from registHER. Clin Cancer Res (2011) 17(14):4834–43. doi: 10.1158/1078-0432.CCR-10-2962 21768129

[5] Hurvitz SA O'Shaughnessy J Mason G Yardley DA Jahanzeb M Brufsky A. Central nervous system metastasis in patients with HER2-positive metastatic breast cancer: patient characteristics, treatment, and survival from SystHERs. Clin Cancer Res (2019) 25(8):2433–41. doi: 10.1158/1078-0432.CCR-18-2366 30593513

[6] Moulder SL Borges VF Baetz T McSpadden T Fernetich G Murthy RK. Phase I study of ONT-380, a HER2 inhibitor, in patients with HER2+–advanced solid tumors, with an expansion cohort in HER2+ metastatic breast cancer (MBC). Clin Cancer Res (2017) 23(14):3529–36. doi: 10.1158/1078-0432.CCR-16-1496 28053022

[7] Pheneger T Bouhana K Anderson D Garrus J Ahrendt K Allen S. Abstract #1795: In Vitro and *in vivo* activity of ARRY-380: a potent, small molecule inhibitor of ErbB2. Cancer Res (2009) 69(9_Supplement):1795.

[8] TUKYSA®Highlights of Prescribing Information, Seagen Inc (2020). US Food and Drug Administration. Available at: https://www.accessdata.fda.gov/drugsatfda_docs/label/2020/213411s000lbl.pdf (Accessed May 2023).

[9] Murthy RK Loi S Okines A Paplomata E Hamilton E Hurvitz SA. Tucatinib, trastuzumab, and capecitabine for HER2-positive metastatic breast cancer. N Engl J Med (2020) 382(7):597–609. doi: 10.1056/NEJMoa1914609 31825569

[10] Gennari A André F Barrios CH Cortés J de Azambuja E DeMichele A. ESMO Clinical Practice Guideline for the diagnosis, staging and treatment of patients with metastatic breast cancer. Ann Oncol (2021) 32(12):1475–95. doi: 10.1016/j.annonc.2021.09.019 34678411

[11] Giordano SH Franzoi MAB Temin S Anders CK Chandarlapaty S Crews JR. Systemic therapy for advanced human epidermal growth factor receptor 2–positive breast cancer: ASCO guideline update. J Clin Oncol (2022) 40(23):2612–35. doi: 10.1200/JCO.22.00519 35640077

[12] Ma X Long L Moon S Adamson BJS Baxi SS. Comparison of population characteristics in real-world clinical oncology databases in the US: Flatiron Health, SEER, and NPCR. medRxiv (2020) 2020. doi: 10.1101/2020.03.16.20037143

[13] Birnbaum B Nussbaum N Seidl-Rathkopf K Agrawal M Estevez M Estola E. Model-assisted cohort selection with bias analysis for generating large-scale cohorts from the EHR for oncology research. ArXiv (2020). doi: 10.48550/arXiv.2001.09765

[14] Walker MS Herms L Miller PJE. Performance of time to discontinuation and time to next treatment as proxy measures of progression-free survival, overall and by treatment group. J Clin Oncol (2020) 38(15_Supplement):e19135. doi: 10.1200/JCO.2020.38.15_suppl.e19135

[15] Campbell BA Scarisbrick JJ Kim YH Wilcox RA McCormack C Prince HM. Time to next treatment as a meaningful endpoint for trials of primary cutaneous lymphoma. Cancers (Basel) (2020) 12(8):2311. doi: 10.3390/cancers12082311 32824427 PMC7463470

[16] Phillips CM Parmar A Guo H Schwartz D Isaranuwatchai W Beca J. Assessing the efficacy-effectiveness gap for cancer therapies: a comparison of overall survival and toxicity between clinical trial and population-based, real-world data for contemporary parenteral cancer therapeutics. Cancer (2020) 126(8):1717–26. doi: 10.1002/cncr.32697 31913522

[17] Nordon C Karcher H Groenwold RHH Ankarfeldt MZ Pichler F Chevrou-Severac H. The "Efficacy-effectiveness gap": historical background and current conceptualization. Value Health (2016) 19(1):75–81. doi: 10.1016/j.jval.2015.09.2938 26797239

[18] Amler N Zottmann D Bierbaum M Schöffski O. Efficacy-Effectiveness gap—extent, causes and implications. Value Health (2015) 18(7):A567. doi: 10.1016/j.jval.2015.09.1864

[19] Sheffield KM Dreyer NA Murray JF Faries DE Klopchin MN. Replication of randomized clinical trial results using real-world data: paving the way for effectiveness decisions. J Comp Eff Res (2020) 9(15):1043–50. doi: 10.2217/cer-2020-0161 32914653

[20] Cramer-van der Welle CM Peters BJM Schramel FMNH Klungel OH Groen HJM van de Garde EMW. Systematic evaluation of the efficacy–effectiveness gap of systemic treatments in metastatic nonsmall cell lung cancer. Eur Respir J (2018) 52(6):1801100. doi: 10.1183/13993003.01100-2018 30487206 PMC6306150

[21] Lakdawalla DN Shafrin J Hou N Peneva D Vine S Park J. Predicting real-world effectiveness of cancer therapies using overall survival and progression-free survival from clinical trials: empirical evidence for the ASCO value framework. Value Health (2017) 20(7):866–75. doi: 10.1016/j.jval.2017.04.003 28712615

[22] Sanoff HK Chang Y Lund JL O'Neil BH Dusetzina SB. Sorafenib effectiveness in advanced hepatocellular carcinoma. Oncologist (2016) 21(9):1113–20. doi: 10.1634/theoncologist.2015-0478 PMC5016063 27185615

[23] Sheikh N Chambers CR. Efficacy vs. effectiveness: erlotinib in previously treated non-small-cell lung cancer. J Oncol Pharm Pract (2013) 19(3):228–36. doi: 10.1177/1078155212464087 23175450

[24] Ethier JL Desautels D Robinson A Amir E Kong W Booth CM. Practice patterns and outcomes of novel targeted agents for the treatment of ERBB2-positive metastatic breast cancer. JAMA Oncol (2021) 7(9):e212140. doi: 10.1001/jamaoncol.2021.2140 34236387 PMC8267844

[25] Cortés J Kim SB Chung WP Im SA Park YH Hegg R. Trastuzumab deruxtecan versus trastuzumab emtansine for breast cancer. N Engl J Med (2022) 386(12):1143–54. doi: 10.1056/NEJMoa2115022 35320644

[26] Referenced with permission from the NCCN Clinical Practice Guidelines in Oncology (NCCN Guidelines®) for Breast Cancer V.4.2023 (2023). National Comprehensive Cancer Network, Inc (Accessed May 18, 2023).

[27] NCT03975647. A study of tucatinib vs. placebo in combination with ado-trastuzumab emtansine (T-DM1) for patients with advanced or metastatic HER2+ breast cancer . US National Library of Medicine: National Institutes of Health. Available at: https://www.clinicaltrials.gov/ct2/show/nct03975647 (Accessed May 2023).

[28] Hamilton EP O'Sullivan CCM Martin M Sohn J Tryfonidis K Santarpia L. Phase 3 study of tucatinib or placebo in combination with trastuzumab and pertuzumab as maintenance therapy for HER2+ metastatic breast cancer (HER2CLIMB-05, trial in progress). J Clin Oncol (2022) 40(16_suppl):TPS1108. doi: 10.1200/JCO.2022.40.16_suppl.TPS1108

