# Supplementary material for: Real-world patient characteristics, treatment patterns, and clinical outcomes associated with tucatinib therapy in HER2-positive metastatic breast cancer
Source: Front Oncol. 2023 Oct 2;13:1264861. doi: 10.3389/fonc.2023.1264861 (PMC10578436; doi:10.3389/fonc.2023.1264861)
Supplement: Supplementary file 1 [file Table_1.docx]

Supplementary Material

Real-world patient characteristics, treatment patterns, and clinical outcomes associated with tucatinib therapy in HER2-positive metastatic breast cancer

Peter A. Kaufman^1^, Edward Neuberger^2*^, Naomi R.M. Schwartz^2^, Shu Wang^3^, Yutong Liu^3^, Ling-I Hsu^2^, Karen Bartley^2^, Matthew T. Blahna^2^, Brian T. Pittner^2^, Gabriel Wong^2^, Carey Anders^4^

^1^Division of Hematology and Oncology, University of Vermont Medical Center, Burlington, VT, United States

^2^Seagen Inc., Bothell, WA, United States

^3^Genesis Research, Hoboken, NJ, United States

^4^Division of Medical Oncology, Duke Cancer Institute, Durham, NC, United States

Supplementary Table 1. Patient selection and attrition in this study from the EHR-derived database

| **Criteria** | **N (% of prior step)** |
| --- | --- |
| Were MBC patients in the EHR-derived database (December 2022 data cut) | 32,819 |
| Were diagnosed on or after January 1, 2017 | 16,733 (50.9%) |
| Possessed evidence of HER2-receptor positivity prior to or within 90 days of index date^a^ | 3,449 (20.6%) |
| Received tucatinib | 255 (7.4%) |
| Had recorded activity within 90 days; aged ≥18 years at index date^a^ | 227 (89.0%) |
| Received systemic anticancer treatment in the metastatic setting following tucatinib | 222 (98%) |
| Had no evidence of other primary cancers in the 6 months prior to index | 217 (97.8%) |
| Had ≥1-day medication supply documented in the EMR | 216 (99.5%) |

^a^Defined as the date of first diagnosis of MBC.

EHR, electronic health record; EMR, electronic medical record; HER2, human epidermal growth factor 2; MBC, metastatic breast cancer.

Supplementary Table 2. Baseline characteristics for patients with HER2+ MBC prior to tucatinib initiation, from the EHR-derived database

| **Characteristic** | **Overall**  **(N=216)** | **Brain metastases prior to treatment**  **(N=153)** | **No brain metastases prior to treatment (N=63)** |
| --- | --- | --- | --- |
| Age (years), median (IQR) | 56 (45, 63) | 53 (44, 62) | 62 (54, 69) |
| Year of therapy initiation, n (%)  2020  2021  2022 | 61 (28.2)  93 (43.1)  62 (28.7) | 43 (28.1)  64 (41.8)  46 (30.1) | 18 (28.6)  29 (46.0)  16 (25.4) |
| ECOG PS, n (%)  0  1  2+  Missing | 65 (30.1)  83 (38.4)  21 (9.7)  47 (21.8) | 40 (26.1)  62 (40.5)  17 (11.1)  34 (22.2) | 25 (39.7)  21 (33.3)  4 (6.3)  13 (20.6) |
| Number of metastatic sites, n (%)  1  2  3+ | 31 (14.4)  51 (23.6)  134 (62.0) | 17 (11.1)  29 (19.0)  107 (69.9) | 14 (22.2)  22 (34.9)  27 (42.9) |
| Sites of metastasis, n (%)  Brain  Bone  Visceral (lung/liver) | 153 (70.8)  137 (63.4)  140 (64.8) | 153 (100)  93 (60.7)  97 (63.4) | 0 (0)  44 (69.8)  43 (68.3) |
| Prior lines of therapy, median (IQR) | 2 (1-3) | 1 (1-3) | 3 (2-4) |
| Prior metastatic cancer treatment exposure, n (%)  T-DXd  T-DM1  Trastuzumab  Pertuzumab  Lapatinib/Neratinib  Chemotherapy | 41 (19.0)  109 (50.5)  167 (77.3)  149 (69.0)  37 (17%)  69 (32%) | 11 (7.2)  63 (41.2)  117 (76.5)  106 (69.3)  26 (17%)  43 (28%) | 30 (47.6)  46 (73.0)  50 (79.4)  43 (68.3)  11 (17%)  26 (41%) |
| Months follow-up, median (IQR) | 12 (6-18) | 12 (6-18) | 12 (5-18) |
| Months to therapy start, median (IQR) | 22 (13-33) | 19 (11-32) | 26 (16-39) |

BM, brain metastases; ECOG PS, Eastern Cooperative Oncology Group performance status; EHR, electronic health record;HER2, human epidermal growth factor 2; MBC, metastatic breast cancer; T-DM1, ado-trastuzumab emtansine; T-DXd, fam-trastuzumab deruxtecan.

**Supplementary Table 3.** Median rwTTD, rwTTNT, and rwOS by LOT among patients with HER2+ MBC receiving tucatinib regimens in the EHR-derived database

|  | **1L** | **2L** | **3L** | **4L+** |
| --- | --- | --- | --- | --- |
| **rwTTD** | | | | |
| All patients (n=216) | n=23 | n=69 | n=44 | n=80 |
| Median, months (95% CI) | 9.1 (5.1-NR) | 8.2 (6.3-18.2) | 9.4 (5.6-13.6) | 4.8 (3.5-7.1) |
| Approved tucatinib triplet combination in any line^a^ (n=145) | - | n=52 | n=31 | n=61 |
| Median, months (95% CI) | - | 8.6 (6.3-18.8) | 9.4 (5.4-15.6) | 5.4 (4.2-8.8) |
| Approved tucatinib triplet combination in 2L and 3L^b^ (n=83) | - | n=83 | | - |
| Median, months (95% CI) | - | 9.4 (6.3-14.1) | | - |
| **rwTTNT** | | | | |
| All patients (n=216) | n=23 | n=69 | n=44 | n=80 |
| Median, months (95% CI) | 12.8 (9.1-NR) | 9.8 (6.5-18.3) | 9.4 (5.7-13.6) | 6.5 (5.1-8.8) |
| Approved tucatinib triplet combination in any line^a^ (n=145) | - | n=52 | n=31 | n=61 |
| Median, months (95% CI) | - | 11.0 (6.8-18.3) | 9.5 (5.4-15.7) | 8.1 (6.2-11.9) |
| Approved tucatinib triplet combination in 2L and 3L^b^ (n=83) | - | n=83 | | - |
| Median, months (95% CI) | - | 9.8 (6.8-14.1) | | - |
| **rwOS** |  |  |  |  |
| All patients (n=216) | n=23 | n=69 | n=44 | n=80 |
| Median, months (95% CI) | 15.5 (13.8-NR) | NR (20.0-NR) | NR | 16.6 (12.2-NR) |
| Approved tucatinib triplet combination in any line^a^ (n=145) | - | n=52 | n=31 | n=61 |
| Median, months (95% CI) | - | 21.3 (17.1-NR) | NR | 16.6 (12.2-NR) |
| Approved tucatinib triplet combination in 2L and 3L^b^ (n=83) | - | n=83 | | - |
| Median, months (95% CI) | - | NR | | - |

^a^Patients who received the FDA-approved tucatinib triplet combination (tucatinib in combination with trastuzumab and capecitabine) after receiving ≥1 HER2-directed therapies in the metastatic setting.

^b^Patients who received the FDA-approved tucatinib triplet combination in 2L or 3L following ≥1 prior HER2-directed therapies in the metastatic setting.

1L, first-line; 2L, second-line; 3L, third-line; 4L+, fourth-line and beyond; EHR, electronic health record; HER2, human epidermal growth factor receptor 2; LOT, line of therapy; MBC, metastatic breast cancer; OS, overall survival; NR, not reached; rw, real world; TTD, time to discontinuation; TTNT, time to next treatment.

**Supplementary Table 4**. Baseline characteristics^a^ for patients with HER2+ MBC in the post–T-DXd tucatinib subgroup from the EHR-derived database

| **Characteristic** | **All patients (n=35)** |
| --- | --- |
| Age (years), median (range) | 59 (36-84) |
| ECOG PS, n (%) | |
| 0 | 12 (34.3) |
| 1 | 15 (42.9) |
| 2+ | 3 (8.6) |
| Missing | 5 (14.3) |
| Sites of metastasis,^b^ n (%) | |
| Bone | 26 (74.3) |
| Lung | 19 (54.3) |
| Liver | 18 (51.4) |
| Brain | 7 (20.0) |
| Prior lines of therapy, median (range) | 3 (1-10) |
| 1 | 2 (5.7) |
| 2 | 3 (8.6) |
| 3+ | 30 (85.7) |
| Treatment exposure in the metastatic setting prior to tucatinib treatment initiation, n (%) | |
| T-DXd | 35 (100) |
| T-DM1 | 30 (85.7) |
| Trastuzumab | 27 (77.1) |
| Pertuzumab | 24 (68.6) |
| Lapatinib/Neratinib | 5 (14%) |
| Chemotherapy | 10 (29%) |

^a^Data for the Flatiron Health database refer to the period prior to tucatinib treatment initiation.
^b^Not mutually exclusive.

ECOG PS, Eastern Cooperative Oncology Group performance status; EHR, electronic health record; HER2, human epidermal growth factor receptor 2; MBC, metastatic breast cancer; T-DM1, ado-trastuzumab emtansine; T-DXd, fam-trastuzumab deruxtecan.
